# Case report: Multiple cryotherapy sessions in localized Darier's disease: a rare clinical presentation and literature review

**DOI:** 10.3389/fmed.2025.1535961

**Published:** 2025-01-30

**Authors:** Yansi Lyu, Bin Wang, Lyuxin Guan, Li Ma, Hao Li

**Affiliations:** ^1^Department of Dermatology, Shenzhen University General Hospital, Shenzhen, Guangdong, China; ^2^Department of Burn and Plastic Surgery, The First Affiliated Hospital of Shenzhen University, Shenzhen, China; ^3^The University of Hong Kong-Shenzhen Hospital, Shenzhen, Guangdong, China

**Keywords:** localized Darier's disease, cryotherapy, the inner thigh, clinical manifestations, therapeutic strategies

## Abstract

Darier's disease (DD), a rare hereditary acantholytic dermatosis with high penetrance but variable expressivity, has about 10% of its cases presenting as localized lesions, known as Localized Darier's Disease (LDD). To date, only a very small number of non-segmental LDD cases have been reported. This study reports a case of a 27-year-old Chinese male with LDD, who presented with yellow-brown papules and plaques on the inner side of the right thigh for 1 month. The diagnosis of localized keratosis follicularis was confirmed through dermatoscopic examination and histopathological assessment of the skin lesion. After repeated applications of cryotherapy combined with the use of emollients, while avoiding physical friction and sun exposure, the rash gradually subsided. The patient experienced no adverse reactions after treatment. The occurrence of LDD on the inner thigh, a relatively rare site, provides new insights into the affected areas of LDD, further expands the clinical manifestations of the disease, and offers valuable experience for clinicians in recognizing and managing LDD at different sites, aiding in the optimization of diagnostic and therapeutic strategies.

## 1 Introduction

Darier's disease (DD), also known as keratosis follicularis, is a rare hereditary acantholytic dermatosis, inherited in an autosomal dominant pattern, caused by mutations in the ATP2A2 [ATPase, Ca(++) transporting, cardiac muscle, slow-twitch 2] gene, with high penetrance but variable expressivity (1). Its clinical features include abnormal keratinization and lack of epidermal cohesion, symmetrically distributed multiple hyperkeratotic papules with pigmentation, mainly involving the seborrheic areas with confluent greasy plaques (2, 3). The prevalence in the general population is about 1/30,000–1/100,000, with equal incidence in men and women, usually manifesting in the second or third decade of life (4, 5).

DD manifests as pruritic, widespread, and symmetrically distributed keratotic papules and plaques, often involving seborrheic areas and skin folds (6). In pigmented skin, it presents as hypopigmented macules distributed around hair follicles (6). A small subset of Darier's disease patients may exhibit localized lesions, including segmental subtypes (1). Histologically, it is characterized by acantholysis with suprabasal clefts, dyskeratotic keratinocytes, corps ronds, and grainy bodies.

Darier's disease secondary infections, such as herpes simplex virus infections, can lead to significant morbidity and mortality (7). Moreover, the quality of life of patients is significantly affected, including impaired self-esteem and impacted social relationships (8). Studies also suggest that DD may have a potential comorbidity with neuropsychiatric diseases (9, 10). In addition to avoiding triggering factors and symptom management, there is currently no effective treatment for DD (11). Although multiple case reports and non-experimental studies have mentioned various methods used to treat the disease, the quality of evidence remains low (11). Identifying targeted therapies for DD is a major concern, with over 70% of participants in the UK eDelphi study group ranking it as a priority translational dermatological research question (12).

Localized Darier's Disease (LDD) was first described by Kreibich in 1906 (13). Approximately 10% of DD cases present with localized lesions (segmental, linear), usually unilateral, and may be distributed along the lines of Blaschko (13). LDD may manifest as band-like, linear, or segmental distributions, and may even be distributed along the lines of Blaschko (13). LDD typically has a later onset and may be associated with HIV infection and radiotherapy (14, 15). To date, only a very small number of non-segmental LDD cases have been reported, occurring in the vulva, areola, inframammary area, sun-exposed areas, and cervix (14). This study reports a case of LDD with an onset site on the inner thigh, which is relatively rare and has reference significance for clinical differential diagnosis and treatment.

## 2 Medical records

The patient is a 27-year-old male who presented to Shenzhen University General Hospital on July 11, 2024, with “yellow-brown papules and plaques on the inner side of the right thigh for one month.” He has a history of good health. The patient's family has no genetic disease history, and he has not experienced any significant discomfort or pruritus since the onset of the disease, nor has he had any history of trauma or special exposure. Laboratory tests showed no significant abnormalities.

**Figure 1:**
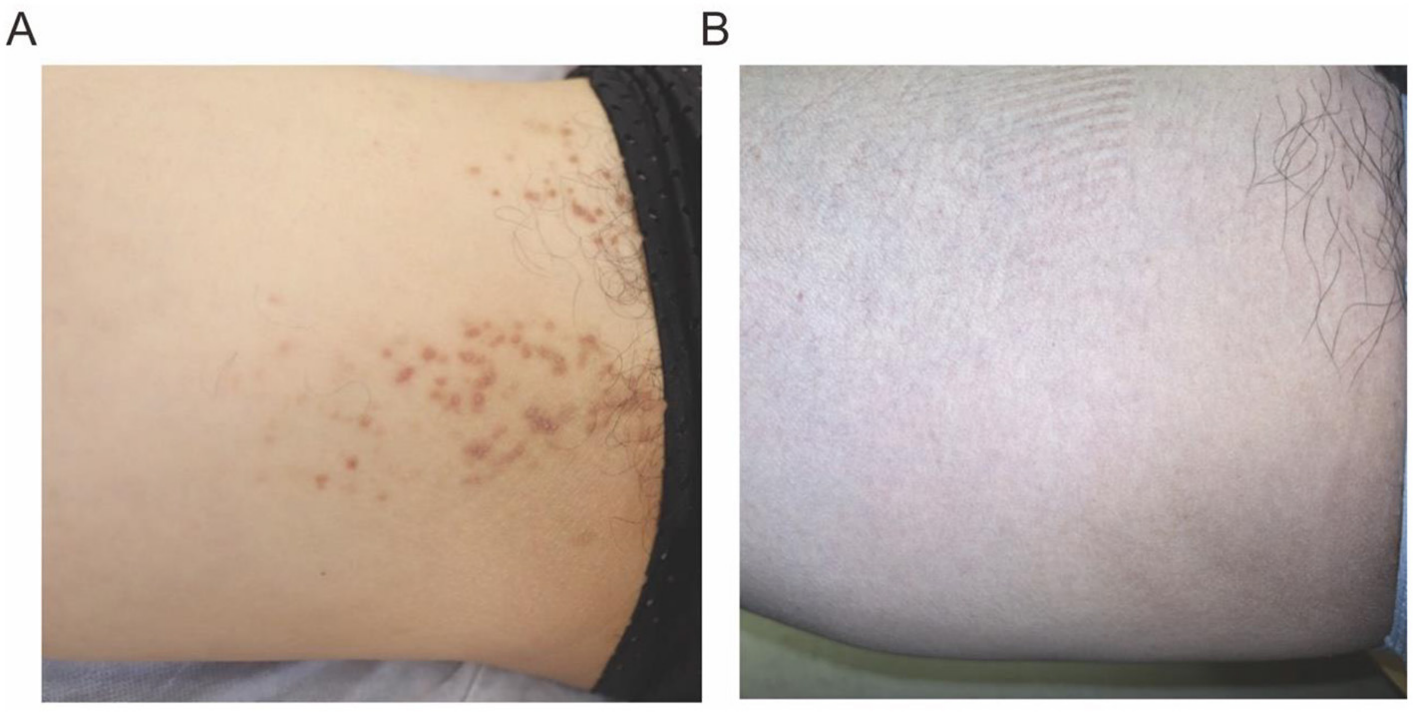
**(A)** Gross examination of the skin reveals yellow-brown warty keratotic papules with a diameter of approximately 3–5 mm, some of which have coalesced to form plaques. **(B)** The follow up image (3 months after the final cryotherapy session).

**Figure 2:**
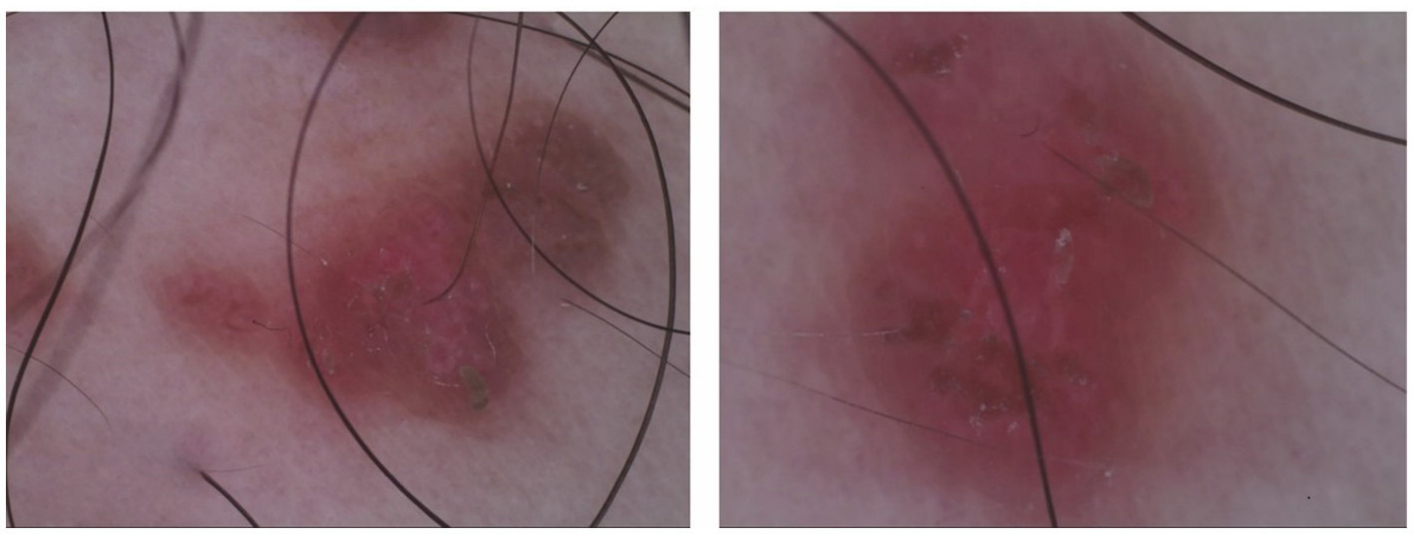
Dermoscopic examination shows varying sizes of circular, stellate, or polygonal yellow-brown areas, with a central white halo surrounded by a pink, structureless area.

On gross examination (Figure 1A), there are yellow-brown warty keratotic papules with a diameter of about 3–5 mm on the inner side of the right thigh, some of which have merged to form plaques. The borders of the lesions are unclear. Dermoscopic examination (Figure 2) reveals circular, star-shaped, or polygonal yellow-brown areas of varying sizes, with a central white halo and surrounded by a pink amorphous area. Some of the central lesions have a yellowish-white pseudocomedo-like structure, with a red depressed erosive surface visible beneath it.

**Figure 3:**
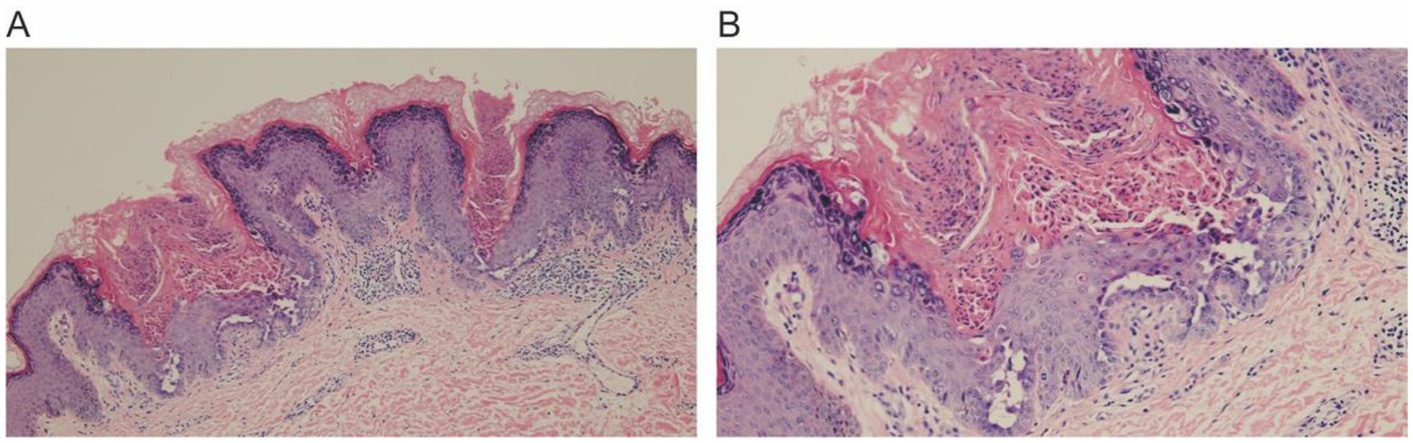
Histopathological examination reveals mild papillomatous changes of the epidermis, associated with acanthosis and thickening of the granular layer. There is focal acantholysis within the epidermis, with dyskeratosis. **(A)** 10×, **(B)** 20×.

The histopathological examination of the skin lesion showed (Figures 3A, B) mild papillomatous changes of the epidermis, with acanthosis and thickening of the granular layer. There is acantholysis within the epidermis, presenting as a focal change, accompanied by dyskeratosis, without any increase in dermal mucin or features of lichen planus, and the final diagnosis was localized keratosis follicularis. Following five biweekly sessions of cryotherapy, complemented by the application of emollients and the avoidance of physical friction and sun exposure, the rash progressively resolved. The post-treatment skin appearance is depicted in Figure 1B. The patient was monitored for a period of 1 year, during which no signs of recurrence were observed.

## 3 Discussion

Genetic skin diseases are considered rare diseases with limited existing knowledge and research, and scientific literature is primarily focused on case reports, lacking large-scale systematic studies (16). DD is caused by mutations in the ATP2A2 gene, which encodes the sarcoplasmic/endoplasmic reticulum calcium ATPase type 2 (SERCA2) protein (3). SERCA2 is responsible for calcium ion transport within the endoplasmic reticulum of epidermal cells, and its dysfunction can lead to abnormal keratinization and cell adhesion defects in the epidermis (5). Approximately 10% of DD lesions exhibit a linear or “band-like” pattern of distribution and may be limited to one side of the body (17). LDD is a type of DD, which was reported by Kreibich in 1906 as a “zosteriform” form of DD (17). O'Malley et al. proposed that localized DD represents a genetic mosaic derived from generalized DD (18). The etiology of LDD (segmental or non-segmental) may be related to cutaneous mosaicism (18, 19). Since DD often presents as hyperkeratotic papules in seborrheic areas, primary care physicians may misdiagnose it, leading to delayed diagnosis and treatment (5). LDD typically presents with mild skin symptoms, and its natural history is generally benign. Traditional DD is usually more severe, often associated with more pronounced keratinization and epidermal dyskeratosis, whereas LDD presents with milder symptoms. Differential diagnoses for segmental DD include Grover's disease (usually transient), Hailey-Hailey disease (mainly affecting skin folds), and autoimmune pemphigus. Therefore, more case reports can help increase dermatologists' awareness of the disease (5).

This study reports a rare case of DD localized to the inner side of the right thigh, where the patient's clinical presentation and laboratory tests lacked specificity, and clinical diagnosis and treatment could only be confirmed through dermatoscopy and pathological examination. Dermatoscopy is an important auxiliary tool for the diagnosis of DD, with typical features including polygonal or circular brown structures (corresponding to hyperkeratosis) and surrounding white halos (corresponding to acantholysis) (20). Errichetti et al. studied 11 patients with biopsy-confirmed extensive DD, describing these consistent features (21). In addition, they observed a uniform pink background, vascular patterns, and white scales. Therefore, dermatoscopic features need to be combined with clinical presentation (2). By combining these dermatoscopic features with clinical manifestations, the diagnostic accuracy of DD can be improved, especially in cases of localized variants, which helps to differentiate it from other similar diseases (21).

Keratosis follicularis must be differentiated from conditions such as seborrheic dermatitis, Hailey-Hailey disease, and Grover's disease (5, 22–26). Seborrheic dermatitis presents with red plaques accompanied by greasy scales, and histological examination shows focal parakeratosis, psoriasis-like acanthosis, and mild to moderate spongiosis (5, 27). Familial benign chronic pemphigus (Hailey-Hailey disease) mainly affects intertriginous areas, with skin lesions characterized by thin-walled, flaccid, inflammatory plaques covered with erosions, crusts, and papillomatous changes, and histological features of “broken brick” acantholysis (5, 23). Grover's disease manifests as small papules or vesicles on the trunk, dermatoscopically appearing as polygonal or stellate yellow/brown areas, similar to DD, with histology showing hyperkeratosis, acanthosis, and mild focal acantholysis, commonly seen in patients with sun damage (5, 24, 25, 28). Additionally, the linear DD in this case must also be distinguished from inflammatory linear verrucous epidermal nevus (ILVEN) and zosteriform herpes (5). The patient presented with yellow to brown verrucous keratotic papules on the inner side of the right thigh, some of which coalesced to form plaques with irregular borders. There were no pruritic symptoms. Dermoscopy revealed yellow to brown areas with white halos. Histopathological examination showed mild acanthotic changes and epidermal dyskeratosis. The final diagnosis was localized follicular keratosis.

Symptoms of DD typically worsen in the summer due to sun exposure, high temperatures, and sweating, which exacerbate the condition (29, 30). Currently, there is no specific treatment for DD, and the treatment goal is to reduce the incidence and prevent complications. Conventional treatment measures include managing infections, moisturizing care, avoiding sun exposure, and mechanical irritation (5). Treatment is primarily symptomatic, combining pharmacological and non-pharmacological therapies. Pharmacological treatment may involve topical and oral retinoids to accelerate cell turnover, as the cell renewal rate is slower in DD patients (31). Topical antimicrobials help prevent secondary infections. Other therapies are less common but include calcineurin inhibitors, synthetic vitamin D analogs, oral corticosteroids, cyclosporine, and low-dose naltrexone (11). Currently, treatment options such as oral acitretin, isotretinoin, systemic vitamin A, topical retinoids, calcipotriol, and tazarotene (with sun protection) are supported by level B evidence (11), while other treatment methods are mostly derived from case reports or case series, with a level C evidence rating (11).

Topical application of 5-fluorouracil has also been shown to be effective in some patients with DD, but its efficacy varies among individuals (1). Given the potential higher risk of neuropsychiatric diseases in patients with DD, it is recommended that patients undergoing oral retinoid treatment be regularly monitored for mood changes (1, 9). Topical diclofenac 3% gel has been described as an effective option for the treatment of DD in prior literature (32). Certain medications such as azathioprine, lithium, and calcium channel blockers may exacerbate the condition and should be used with caution (33). Adjunctive treatment options include dermabrasion, CO2 laser, Er-YAG laser, and surgical excision (11). For patients unresponsive to conventional therapies, surgical treatment may be considered, but currently, dermatologists lack evidence-based guidelines for the treatment of DD (5). After discontinuation of treatment and in the presence of triggering factors such as trauma, high temperature, or sun exposure, both DD and LDD may experience exacerbation or recurrence at the same site (17, 34, 35). Patients should avoid triggering factors such as ultraviolet radiation, high temperatures, and friction.

After literature review, no cases of LDD on the inner side of the thigh have been found. This case is unique and provides new insights into the affected sites of LDD, further expanding the clinical manifestations of the disease. It offers valuable experience for clinicians in recognizing and managing LDD at different sites, aiding in the optimization of diagnostic and therapeutic strategies.

## Data Availability

The original contributions presented in the study are included in the article/supplementary material, further inquiries can be directed to the corresponding authors.
